# Supplementary material for: Unveiling the Bioactive Potential of the Invasive Jellyfish Phyllorhiza punctata Through Integrative Transcriptomic and Proteomic Analyses
Source: Biomolecules. 2025 Aug 4;15(8):1121. doi: 10.3390/biom15081121 (PMC12383608; doi:10.3390/biom15081121)
Supplement: Supplementary file 1 [file biomolecules-15-01121-s001.zip › Suplementary Material/FileS3/PEPFOLD4/XP_065058275_8064/clusters.html]

 PEP-FOLD clusters


Download archive of all models  here   

|  |  |  |
| --- | --- | --- |
| # | file | sOPEP |
| # cluster 1 - Size: 28 |  |  |
|  | XP\_065058275\_8064-model1 | -16.0316 |
|  | XP\_065058275\_8064-model1.1 | -16.0316 |
|  | XP\_065058275\_8064-model1.2 | -16.0316 |
|  | XP\_065058275\_8064-model1.3 | -16.0316 |
|  | XP\_065058275\_8064-model1.4 | -16.0316 |
|  | XP\_065058275\_8064-model1.5 | -16.0316 |
|  | XP\_065058275\_8064-model1.6 | -16.0316 |
|  | XP\_065058275\_8064-model1.7 | -16.0316 |
|  | XP\_065058275\_8064-model1.8 | -16.0316 |
|  | XP\_065058275\_8064-model1.9 | -16.0316 |
|  | XP\_065058275\_8064-model1.10 | -16.0316 |
|  | XP\_065058275\_8064-model1.11 | -16.0316 |
|  | XP\_065058275\_8064-model1.12 | -16.0316 |
|  | XP\_065058275\_8064-model1.13 | -16.0316 |
|  | XP\_065058275\_8064-model1.14 | -16.0316 |
|  | XP\_065058275\_8064-model1.15 | -16.0316 |
|  | XP\_065058275\_8064-model1.16 | -16.0316 |
|  | XP\_065058275\_8064-model1.17 | -16.0316 |
|  | XP\_065058275\_8064-model1.18 | -16.0316 |
|  | XP\_065058275\_8064-model1.19 | -16.0316 |
|  | XP\_065058275\_8064-model1.20 | -16.0316 |
|  | XP\_065058275\_8064-model1.21 | -16.0316 |
|  | XP\_065058275\_8064-model1.22 | -16.0316 |
|  | XP\_065058275\_8064-model1.23 | -15.872 |
|  | XP\_065058275\_8064-model1.24 | -15.842 |
|  | XP\_065058275\_8064-model1.25 | -15.842 |
|  | XP\_065058275\_8064-model1.26 | -15.842 |
|  | XP\_065058275\_8064-model1.27 | -15.842 |
| # cluster 2 - Size: 26 |  |  |
|  | XP\_065058275\_8064-model2 | -16.0084 |
|  | XP\_065058275\_8064-model2.1 | -16.0084 |
|  | XP\_065058275\_8064-model2.2 | -16.0084 |
|  | XP\_065058275\_8064-model2.3 | -16.0084 |
|  | XP\_065058275\_8064-model2.4 | -16.0084 |
|  | XP\_065058275\_8064-model2.5 | -16.0084 |
|  | XP\_065058275\_8064-model2.6 | -16.0084 |
|  | XP\_065058275\_8064-model2.7 | -16.0084 |
|  | XP\_065058275\_8064-model2.8 | -16.0084 |
|  | XP\_065058275\_8064-model2.9 | -16.0084 |
|  | XP\_065058275\_8064-model2.10 | -16.0084 |
|  | XP\_065058275\_8064-model2.11 | -16.0084 |
|  | XP\_065058275\_8064-model2.12 | -16.0084 |
|  | XP\_065058275\_8064-model2.13 | -16.0084 |
|  | XP\_065058275\_8064-model2.14 | -16.0084 |
|  | XP\_065058275\_8064-model2.15 | -16.0084 |
|  | XP\_065058275\_8064-model2.16 | -16.0084 |
|  | XP\_065058275\_8064-model2.17 | -16.0084 |
|  | XP\_065058275\_8064-model2.18 | -16.0084 |
|  | XP\_065058275\_8064-model2.19 | -16.0084 |
|  | XP\_065058275\_8064-model2.20 | -16.0084 |
|  | XP\_065058275\_8064-model2.21 | -15.8188 |
|  | XP\_065058275\_8064-model2.22 | -15.8188 |
|  | XP\_065058275\_8064-model2.23 | -15.8188 |
|  | XP\_065058275\_8064-model2.24 | -15.8188 |
|  | XP\_065058275\_8064-model2.25 | -15.8188 |
| # cluster 3 - Size: 7 |  |  |
|  | XP\_065058275\_8064-model3 | -15.928 |
|  | XP\_065058275\_8064-model3.1 | -15.928 |
|  | XP\_065058275\_8064-model3.2 | -15.928 |
|  | XP\_065058275\_8064-model3.3 | -15.928 |
|  | XP\_065058275\_8064-model3.4 | -15.928 |
|  | XP\_065058275\_8064-model3.5 | -15.928 |
|  | XP\_065058275\_8064-model3.6 | -15.7755 |
| # cluster 4 - Size: 13 |  |  |
|  | XP\_065058275\_8064-model4 | -15.8859 |
|  | XP\_065058275\_8064-model4.1 | -15.8859 |
|  | XP\_065058275\_8064-model4.2 | -15.8859 |
|  | XP\_065058275\_8064-model4.3 | -15.8859 |
|  | XP\_065058275\_8064-model4.4 | -15.8859 |
|  | XP\_065058275\_8064-model4.5 | -15.8859 |
|  | XP\_065058275\_8064-model4.6 | -15.8859 |
|  | XP\_065058275\_8064-model4.7 | -15.8859 |
|  | XP\_065058275\_8064-model4.8 | -15.8859 |
|  | XP\_065058275\_8064-model4.9 | -15.8859 |
|  | XP\_065058275\_8064-model4.10 | -15.8859 |
|  | XP\_065058275\_8064-model4.11 | -15.8859 |
|  | XP\_065058275\_8064-model4.12 | -15.8859 |
| # cluster 5 - Size: 8 |  |  |
|  | XP\_065058275\_8064-model5 | -15.8786 |
|  | XP\_065058275\_8064-model5.1 | -15.8786 |
|  | XP\_065058275\_8064-model5.2 | -15.8786 |
|  | XP\_065058275\_8064-model5.3 | -15.8786 |
|  | XP\_065058275\_8064-model5.4 | -15.8786 |
|  | XP\_065058275\_8064-model5.5 | -15.8786 |
|  | XP\_065058275\_8064-model5.6 | -15.8786 |
|  | XP\_065058275\_8064-model5.7 | -15.8786 |
| # cluster 6 - Size: 14 |  |  |
|  | XP\_065058275\_8064-model6 | -15.8553 |
|  | XP\_065058275\_8064-model6.1 | -15.8553 |
|  | XP\_065058275\_8064-model6.2 | -15.8553 |
|  | XP\_065058275\_8064-model6.3 | -15.8553 |
|  | XP\_065058275\_8064-model6.4 | -15.8553 |
|  | XP\_065058275\_8064-model6.5 | -15.8553 |
|  | XP\_065058275\_8064-model6.6 | -15.8553 |
|  | XP\_065058275\_8064-model6.7 | -15.8553 |
|  | XP\_065058275\_8064-model6.8 | -15.8553 |
|  | XP\_065058275\_8064-model6.9 | -15.8553 |
|  | XP\_065058275\_8064-model6.10 | -15.8553 |
|  | XP\_065058275\_8064-model6.11 | -15.8553 |
|  | XP\_065058275\_8064-model6.12 | -15.8553 |
|  | XP\_065058275\_8064-model6.13 | -15.6258 |
| # cluster 7 - Size: 1 |  |  |
|  | XP\_065058275\_8064-model7 | -15.7701 |
| # cluster 8 - Size: 3 |  |  |
|  | XP\_065058275\_8064-model8 | -15.7545 |
|  | XP\_065058275\_8064-model8.1 | -15.7545 |
|  | XP\_065058275\_8064-model8.2 | -15.7545 |
|  |  |  |
